# Obituary

**Published:** 1909-10-16

**Authors:** 


### OBITUARY.

Surgeon-General Thomas Tarrant, of Charlestown, Queenstown, Cork, Honorary Physician to the King, who died on February 3, aged seventy-nine, left personal estate valued at £4,126, of which £1,390 is English estate.

We have to record the death, at Wimbledon, of Colonel George McBride Davis, C.B., D.S.O., M.D., I.M.S., who had retired from practice.

We have to record the death, at Hove, of Brigade-Surgeon W. P. Ward, late Royal Artillery, in his 85th year. He took his diploma in 1846, and has been on the retired list for some years.

Sir Arthur Mitchell, K.C.B., M.D., LL.D., died on Tuesday evening in the eighty-fourth year of his age at his residence in Edinburgh. Sir Arthur was connected with the Scottish Lunacy Board from its establishment in 1857, and became a Commissioner in Lunacy in 1870, retiring in 1895. He was a member of English and Irish Lunacy Commissions, and may be said to have established the system of caring for the insane in private dwellings, as it at present exists in Scotland. He was vive-president of the Edinburgh Royal Society, and formerly Professor of Ancient History at the Scottish Academy.

The death is announced of Dr. George Joseph Cooper, M.P., who was taken ill on returning to his house in Southwark Park Road early on October 7 after leaving the House of Commons, and died at eleven o'clock the same evening from a stroke of paralysis at the age of 65. Dr. Cooper was educated at the Leeds Grammar School and afterwards at Manchester and University College, London. He took his diploma as member of the Royal College of Surgeons, England, in 1867, and became a licentiate of the Society of Apothecaries four years later. He was formerly resident medical officer of the Poplar Hospital for Accidents and the Bristol General Hospital. He was a member of the London County Council from 1888 to 1906, and had been chairman of the Public Health Committee for six years. In 1906 he was elected member of Parliament for Bermondsey by a majority of 1,759 over Mr. H. J. C. Cust, the Conservative candidate.

